# Combination of Composite Autonomic Symptom Score 31 and Heart Rate Variability for Diagnosis of Cardiovascular Autonomic Neuropathy in People with Type 2 Diabetes

**DOI:** 10.1155/2020/5316769

**Published:** 2020-10-30

**Authors:** Zhiyin Zhang, Yujin Ma, Liujun Fu, Liping Li, Jie Liu, Huifang Peng, Hongwei Jiang

**Affiliations:** ^1^Department of Endocrinology and Metabolism, The First Affiliated Hospital, and College of Clinical Medicine of Henan University of Science and Technology, Luoyang, China; ^2^Clinical Medicine Research Center of Endocrine and Metabolic Diseases of Luoyang, Luoyang, China

## Abstract

**Objective:**

Cardiovascular autonomic neuropathy (CAN) is a common but severe problem of diabetes, which a timely diagnosis may have important clinical implications. This study was carried out to investigate the diagnostic performance of Composite Autonomic Symptom Score 31 (COMPASS 31) combined with heart rate variability (HRV) for cardiovascular autonomic neuropathy in type 2 diabetes.

**Methods:**

A total of 103 hospitalized subjects with type 2 diabetes were recruited in the study. All cases received clinical data collection, laboratory examination, and related complication examinations. Cardiovascular autonomic function was assessed using CARTs, COMPASS 31, and HRV analyses. A score of at least 2 based on CARTs was defined as CAN.

**Results:**

Of the 103 subjects with type 2 diabetes, 41.8% were diagnosed with confirmed CAN. Participants with CAN had considerably higher COMPASS 31 scores. The CAN group showed a significant decrease in all HRV indices. COMPASS 31 scores and HRV indices were closely correlated with CARTs (*P* < 0.05). Receiver operating characteristics (ROC) curve results showed that COMPASS 31 score identified CAN with an AUC value of 0.816, while the AUC values of HRV indices were 0.648 to 0.919, among which SDNN and LF had the best diagnostic value, with the AUC values of 0.919 and 0.865, respectively. When combining COMPASS 31 score with SDNN and LF, the AUC value increased to 0.958, with a sensitivity of 90.7% and a specificity of 86.7%.

**Conclusions:**

The combination of COMPASS 31 and HRV could improve the diagnostic performance of CAN in type 2 diabetes, which might be conducive to the diagnosis of CAN.

## 1. Introduction

Diabetic autonomic neuropathy (DAN) is one of the most common chronic complications of diabetes, which may affect cardiovascular, gastrointestinal, urogenital, and sudomotor function [1]. Among the various forms of DAN, cardiovascular autonomic neuropathy (CAN) is the most severe and studied form. CAN is defined as the impairment of autonomic control of the cardiovascular system in the setting of diabetes after exclusion of other causes [2]. Screening for CAN has been recommended at the diagnosis of type 2 diabetes even if there are no symptoms, particularly in people with a history of poor glycemic control, increased cardiovascular risk, and presence of macrovascular or microvascular complications [2].

In type 2 diabetes, the prevalence of CAN varies widely from 15.5 to 73% [3–5]. The major clinical manifestations of CAN include resting tachycardia, orthostatic hypotension, and exercise intolerance.

CAN is an independent risk factor for any cardiovascular events in people with diabetes, such as arrhythmia and painless myocardial ischemia [6, 7]. At present, cardiovascular reflex tests (CARTs) are regarded as the gold standard in the diagnosis of CAN. However, this method is cumbersome and requires the active cooperation of people, which limits the widespread application in clinical practice. Given that strengthening multifactor intervention can delay the occurrence and development of CAN, a simple and effective tool for CAN is needed to assist with early detection and management of CAN, which is of great significance for improving the prognosis and quality of life in people with diabetes [8].

Composite Autonomic Symptom Score 31 (COMPASS 31) is a comprehensive questionnaire-based scale proposed by Sletten et al. [9] in 2012 to assess the autonomic symptoms across multiple domains. It is proven to be a refined, internally consistent, and quantitative assessment tool of autonomic symptoms. COMPASS 31 has been utilized to evaluate autonomic dysfunction in people with systemic sclerosis, parkinsonism, fibromyalgia, and small-fiber polyneuropathy [10–12]. Moreover, COMPASS 31 was validated in a cohort of people with CAN [13]. A recent study that assessed the diagnostic performance of COMPASS 31 and electrochemical skin conductance found that the combination of the tests can provide a better diagnostic performance for CAN [14].

Heart rate variability (HRV) analysis is widely used in detecting CAN, which can quantitatively assess the tension of sympathetic and parasympathetic nerve and the balance of the two through the measurement and analysis from electrocardiography recordings. It is recommended by the Toronto Consensus Panel on Diabetic Neuropathy and the American Diabetes Association [2, 15] and has been used as the evaluation indicator of CAN in some major clinical trials [16, 17]. Several studies have shown that HRV analysis may be more sensitive and accurate than traditional CARTs in the early detection of CAN [18, 19].

At present, there has been no study on the diagnostic value of COMPASS 31 score combined with HRV analysis in people with type 2 diabetes. Therefore, this study is aimed at using both methods to evaluate the diagnostic performance for CAN in type 2 diabetes, and we hypothesized that the combination of COMPASS 31 score and certain indices of HRV could contribute to the diagnosis of CAN.

## 2. Materials and Methods

### 2.1. Study Subjects

This was a cross-sectional study. From October 2018 to September 2019, participants with type 2 diabetes, aged 18–75 years, were recruited, who were admitted to the Department of Endocrinology of the First Affiliated Hospital of Henan University of Science and Technology. The exclusion criteria were as follows: (1) type 1 diabetes or other types of diabetes; (2) severe cardiovascular diseases, such as myocardial infarction, heart failure, or arrhythmia; (3) acute complications, such as diabetic ketoacidosis (DKA), and a hyperosmolar hyperglycemic state (HHS); (4) acute stroke, severe infection, recent surgery; (5) severe anemia, thyroid disease, and severe liver or kidney dysfunction; (6) mental illness or neurosis; (7) taking drugs that affect heart rate within a month; (8) pregnant or lactating women; and (9) proliferative diabetic retinopathy.

In total, 112 participants were screened for CAN. Among them, 9 participants were excluded: 6 participants did not receive all the examinations and the other 3 participants did not have their CARTs data analyzed. Finally, a total of 103 participant observations were available for the analyses. The study was approved by the Ethics Committee of The First Affiliated Hospital of Henan University of Science and Technology and was performed in accordance with the Declaration of Helsinki. All subjects provided written informed consent to participate.

### 2.2. Methods

#### 2.2.1. Collection of Clinical Data

Complete clinical history and anthropometric data were measured. The data included gender, age, duration of diabetes, smoking history, drinking history, hypertension, and family history of diabetes. Body mass index (BMI), waist-hip ratio (WHR), resting heart rate, and blood pressure were measured.

All biochemical measures were analyzed from venous blood samples (following a minimum of an 8-hour fast) except for urinary albumin and creatinine which were measured from urine samples. Biochemical indicators included glycated hemoglobin (HbA1c), fasting blood glucose (FBG), triglyceride (TG), total cholesterol (TC), high-density lipoprotein cholesterol (HDL-c), and low-density lipoprotein cholesterol (LDL-c). HbA1c was measured by high-performance liquid chromatography. FBG, TG, TC, HDL-c, and LDL-c were detected by the Siemens ADVIA 2400 automatic biochemical analyzer. Urinary albumin and creatinine levels were measured on a random urine sample by an enzyme immunoassay to evaluate renal function using the urinary albumin-to-creatinine ratio (UACR).

Distal symmetric polyneuropathy (DSPN) was confirmed by nerve conduction studies (NCS), and the exclusion of neuropathy by other causes [1]. For each subject, NCS were assessed with an electromyography (EMG) apparatus (Haishen NDI-097, Shanghai, China) by a professional physician in the electromyography room. NCS were performed on median, ulnar, peroneal, and tibial nerves for motor conduction velocity, and median, ulnar, and sural nerves for sensory conduction velocity. Slowness in the motor or sensory conduction velocity on two or more nerves less than the normal limit (mean − 2 SD) were identified as DSPN [20]. Diabetic nephropathy (DN) was defined as the presence of albuminuria (≥30 mg/g of UACR). Based on the Early Treatment Diabetic Retinopathy Study (ETDRS) retinopathy severity scale [21], diabetic retinopathy (DR) was evaluated and graded by an experienced ophthalmologist as no apparent retinopathy, nonproliferative diabetic retinopathy, and proliferative diabetic retinopathy through an optical fundus camera (Canon CR-2, Tokyo, Japan).

#### 2.2.2. Cardiovascular Reflex Tests (CARTs)

Standardized cardiovascular reflex tests were performed by the recording of a continuous electrocardiogram as previously described [22], which included tests of heart rate responses, such as heart rate responses to deep breathing, to Valsalva maneuver, and to lying to standing (30 : 15 ratio). The three tests explore mainly on the parasympathetic function, but the nervous pathways and reflex mechanisms involved are not identical: Valsalva maneuver and 30 : 15 ratio involves both sympathetic and parasympathetic arms [23, 24]. Data were obtained by a 12-lead electrocardiography using MedEx ECG-2000 network system (MedEx, Beijing, China). Another test was the blood pressure responses to lying to standing (postural BP change) which assessed sympathetic function. It was measured using an electronic sphygmomanometer (Omron HEM-7136, Kyoto, Japan). Subjects were asked to have a rest at least 20 minutes before the tests and avoid intake of caffeine, smoking, alcohol, and food at least 2 h before testing. There were 5-minute intervals between each test, and all tests were performed by the same physician. Each of the four tests described above was scored as 0 for normal, 0.5 for borderline, and 1 for abnormal, for a total score of 4. Age-related normal reference values were used to define abnormality. The participants with a score of at least 2 was defined as CAN group and those less than 2 as non-CAN group [2, 22].

#### 2.2.3. Composite Autonomic Symptom Score 31 (COMPASS 31)

All subjects were requested to complete COMPASS 31 questionnaire independently according to the actual situation. All questionnaires were administered by the same physician. COMPASS 31 comprises 6 domains with 31 items (orthostatic intolerance 4 items, vasomotor 3 items, secretomotor 4 items, gastrointestinal 12 items, bladder 3 items, and pupillomotor 5 items) and provides the minimal weighted total score equals 0 and the maximum weighted total score equals 100. The higher the score, the more severe the autonomic neuropathy [9].

#### 2.2.4. Heart Rate Variability (HRV) Analysis

For the HRV analysis, the ECG data were monitored continuously by 24-hour Holter recordings with BENEWARE Smart Ambulatory Electrocardiogram Analysis System (Zhejiang, China). And the completed data were analyzed by an experienced physician. Time domain analysis and frequency domain analysis were obtained. The selected time domain indices were the standard deviation (SD) of the NN intervals (SDNN), the percentage of adjacent NN intervals with a difference greater than 50 ms (PNN50), and the root mean square differences of successive NN intervals (RMSSD). For the frequency domain analysis, low-frequency (LF) and high-frequency (HF) powers were assessed, as well as the LF/HF ratio [25].

### 2.3. Statistical Analysis

Statistical analyses were performed using SPSS software (version 24.0, Chicago, IL) and MedCalc software (version 15.2.2, Ostend, Belgium). The Kolmogorov-Smirnov test was used to determine whether continuous variables followed a normal distribution. Normally distributed continuous variables were expressed as means ± standard deviation (SD), whereas variables with skewed continuous distribution were expressed as median (interquartile range). Differences in continuous variables between groups were assessed by *t*-test and Mann-Whitney *U* test. Categorical variables were expressed as number (percentage), and a chi-square test was used to compare different groups. Associations between variables were assessed using Spearman's rank correlation. We conducted receiver operating characteristic (ROC) analyses to evaluate the diagnostic performance of COMPASS 31 score and HRV indices, while the combination of them was evaluated by established multivariable-adjusted logistic regression mode. Kappa test was also used to analyze the consistency between each variable and gold standard.

## 3. Results

### 3.1. Participant Characteristics

The characteristics of 103 participants with type 2 diabetes are presented in Table 1. The participants consisted of 37 female (35.9%) and 66 male (64.1%), a mean age of 54 ± 9 years, and a median diabetes duration of 8(3, 15) years. A total of 43 participants (41.8%) were diagnosed with CAN. Participants with CAN were older with a longer duration of diabetes, increased resting heart rate, and elevated UACR (*P* < 0.05). In addition, the rates of DSPN, DN, and DR were also significantly higher among those with CAN.

### 3.2. Cardiovascular Autonomic Nervous Function

As demonstrated in Table 2, abnormal CARTs including deep breathing, lying to standing (30 : 15 ratio), the Valsalva maneuver, and postural BP change were shown in 65 subjects (63.1%), 14 subjects (13.6%), 37 subjects (35.9%), and 6 subjects (5.8%), respectively. The COMPASS 31 scores differed significantly between participants with and without CAN (*P* < 0.001). Furthermore, we compared domain scores, participants with CAN had significantly higher scores for orthostatic intolerance score (*P* < 0.001), vasomotor score (*P* = 0.01), gastrointestinal score (*P* < 0.001), bladder score (*P* = 0.001) (see Table S1). For HRV analysis, all indices in participants with CAN were significantly lower than those without CAN.

### 3.3. Association of COMPASS 31 Score, HRV Indices, and CARTs

A significant positive association was found between COMPASS 31 score and CARTs score (*r*_s_ = 0.547, *P* < 0.001), while each HRV index was significantly inversely related to CARTs score (*r*_s_ = −0.284~−0.722, *P* < 0.01). Among these indices of HRV, SDNN (*r*_s_ = −0.722, *P* < 0.001) and LF (*r* = −0.637, *P* < 0.001) had the strongest correlation with CARTs score. In addition, the COMPASS 31 score was negatively correlated with SDNN, LF and LF/HF (*r*_s_ = −0.222~−0.397, *P* < 0.05) (Table 3).

### 3.4. Diagnostic Performance of COMPASS 31 and HRV Indices for CAN

**Figure 1:**
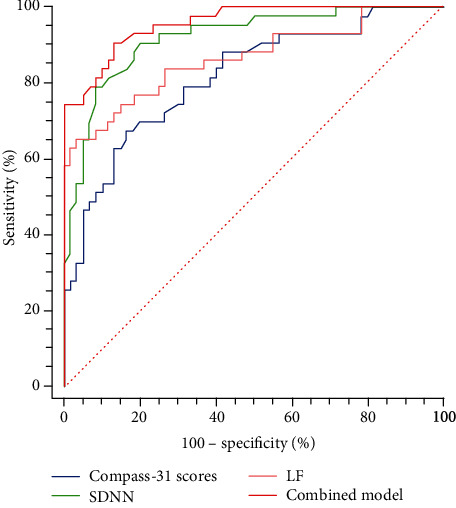
ROC curves comparing the ability of different variables and the combined model in distinguishing between participants with and without CAN. Combined model: the combination of COMPASS 31 score, SDNN, and LF.

We explored the diagnostic value of COMPASS 31 score and HRV indices for CAN by using CARTs as the gold standard (Table 4, Figure 1). COMPASS 31 score showed a fair diagnostic value with the AUC of 0.816, and the cutoff was 19.5 with sensitivity of 67.4% and specificity of 83.3%. When considering the diagnostic performance of HRV indices, the AUC values of HRV indices for diagnosing CAN were 0.648 to 0.919, among which SDNN and LF had significantly higher diagnostic value than other indices (*P* < 0.05), with the AUC value of 0.919 and 0.865, respectively. However, there was no significant difference of diagnostic value between these two indices. The optimal cutoff of SDNN for diagnosing CAN was 95 ms, with sensitivity of 79.1% and specificity of 91.7%, while of LF was deemed to be 131.4 ms^2^ for CAN (sensitivity of 65.1%, specificity of 96.7%). The diagnosis model incorporating COMPASS 31 score, SDNN, and LF was further analyzed. The AUC value for the combined model could be increased to 0.958, with sensitivity of 90.7%, specificity of 86.7%, and the Kappa value increased to 0.75.

## 4. Discussion

Dysfunction of the autonomic nervous system in CAN cause impaired cardiovascular regulation, resulting in an increased risk of cardiovascular death in patients with type 2 diabetes mellitus. Autonomic dysfunction plays an important role in the occurrence of arrhythmic events as ventricular arrhythmias and atrial fibrillation [26, 27]. Indeed, a method that is easy and noninvasive for diagnosis of CAN is necessary, for the timely treatment and a reduced risk of cardiovascular events. In the present study, we first explored the diagnostic performance of the combination of COMPASS 31 score and HRV indices for CAN in type 2 diabetes. Our data observed that a combination of COMPASS 31 score, SDNN, and LF showed a greater diagnostic ability than using COMPASS 31 score or HRV indices alone, while with a high sensitivity and specificity. For the diagnosis of CAN, autonomic function test is particularly important, and the evaluation of autonomic symptoms cannot be ignored as well. Therefore, a combination of both may provide a better diagnostic method for CAN in clinical practice.

Autonomic symptoms should be assessed in people with diabetes as recommended by current guidelines [15]. We evaluated autonomic symptoms in participants with type 2 diabetes by using COMPASS 31; the data showed that COMPASS 31 score differed significantly between participants with CAN and without CAN. Each subscale score was also higher than participants without CAN, in particular for orthostatic intolerance, vasomotor, gastrointestinal, and bladder symptoms. This finding confirms the observation in the previous studies [28, 29] that CAN may be closely related to autonomic dysfunction of other organs. Therefore, participants with other types of autonomic symptoms should be alert to the occurrence of CAN in clinic. We also found that COMPASS 31 score increased with the decrease of HRV indices, and a similar finding was obtained in a study of people with fibromyalgia [10]. In addition, the diagnostic performance of COMPASS 31 seemed to be better than that observed in similar studies [13, 30], and the cutoff was higher as well. We speculated that there be difference of the sample size, study subjects, and racial differences between studies, leading to different results. Nevertheless, what we have in common is that COMPASS 31 could be an effective tool for CAN.

HRV analysis is considered to be a widely used and readily available diagnostic method. It includes time domain and frequency domain analysis. Among the time domain indices, SDNN mainly reflects the change of overall HRV, while RMSSD and PNN50 represent the parasympathetic activity. LF and HF assess the sympathetic and parasympathetic functions, respectively, as frequency domain indices, and LF/HF ratio can be used to evaluate the interaction of both [17, 31]. Compared with subjects without CAN, we found that those with CAN had significantly reduced overall HRV, including the loss of parasympathetic and sympathetic, with mainly decreased parasympathetic. It indicated that parasympathetic impairment may precede the sympathetic dysfunction. Actually, the association between type 2 diabetes mellitus and autonomic dysfunction is not well established and still under investigation. A prospective multicenter study has shown that the existing link between autonomic dysfunction and the increase and recurrence of vaso vagal syncope events in type 2 diabetes mellitus might be the result of the excess in parasympathetic tone in contrast to sympathetic heart innervations [32]. Furthermore, a good correlation has been shown between HRV indices and CARTs. It is worth reminding that SDNN and LF have the best consistency with CARTs, which showed the highest diagnostic values. A study by Viggiano et al. [33], which observed that autonomic dysfunction could be detected by LF in participants with type 2 diabetes without obvious symptoms. SDNN, as an important indicator of HRV analysis, reflects the total tension of autonomic activity, with good repeatability and stability, which has been often used as a predictor of the prognosis in cardiovascular event [34]. Hence, SDNN and LF seem to be used as the important indicators for assessing CAN clinically. In the current study, the obtained cutoff values of HRV indices were lower than the values proposed in a study conducted in 1996 [25], while its interpretation and application need to be further confirmed by multicenter and large-scale studies.

Some limitations of the present study deserved to be noted. First, this is a single -center and cross-sectional study, resulting in a relatively small sample size, a limited scope of inclusion, and no relevant analysis by the severity of CAN. Second, the study subjects are all in-patients, leading to these results not being applicable to the out-patient population. Third, control subjects were not set in this study, because the primary aim was to evaluate the diagnostic capacity of COMPASS 31 and HRV indices for CAN. A large multicenter and longitudinal sample study still requires to be further confirmed.

## 5. Conclusions

In the present study, we found that COMPASS 31 and HRV indices were in good agreement with conventional CARTs, and the selected HRV indices SDNN and LF might be used as the important reference indicators. The combination of COMPASS 31 and HRV analysis is conducive to the diagnosis of CAN, providing certain reference for clinical practice. The limited scope of inclusion of this study requires further validation in the out-patient population with diabetes.

## Figures and Tables

**Table 1:** Clinical characteristics of the study population.

Variable	Overall (*n* = 103)	Non-CAN (*n* = 60)	CAN (*n* = 43)	*P* value
Female, *n*(%)	37(35.9)	18(30.0)	19(44.2)	0.139
Age (years)	54 ± 9	51 ± 9	58 ± 8	<0.001^∗∗∗^
Duration of diabetes (years)	8(3, 15)	5(2, 10)	15(6, 18)	<0.001^∗∗∗^
Smoking history, *n*(%)	24(23.3)	12(20.0)	12(27.9)	0.349
Drinking history, *n*(%)	23(22.3)	15(25.0)	8(18.6)	0.442
Hypertension, *n*(%)	49(47.6)	31(51.7)	18(41.9)	0.326
Family history of diabetes, *n*(%)	45(43.7)	27(45.0)	18(41.9)	0.751
Body mass index (kg/m^2^)	24.6 ± 2.9	24.5 ± 2.7	24.5 ± 3.2	0.947
Waist-hip ratio	0.95 ± 0.06	0.94 ± 0.06	0.95 ± 0.06	0.556
Resting heart rate (bpm)	73 ± 11	70 ± 10	76 ± 13	0.013^∗^
Blood pressure (mmHg)				
Systolic BP	127 ± 15	126 ± 15	128 ± 16	0.508
Diastolic BP	81 ± 10	81 ± 10	80 ± 9	0.404
Microvascular complications, *n*(%)				
DSPN	67(65.0)	32(53.3)	35(81.4)	0.003^∗∗^
Diabetic nephropathy	43(41.7)	19(31.7)	24(55.8)	0.014^∗^
Diabetic retinopathy	31(30.1)	9(15.0)	22(51.2)	<0.001^∗∗∗^
Laboratory variables				
Fasting blood glucose (mmol/L)	9.8 ± 3.3	9.7 ± 3.6	9.9 ± 3.0	0.764
HbA1c (%)	9.6 ± 2.7	9.6 ± 2.9	9.7 ± 2.5	0.864
Total cholesterol (mmol/L)	4.76 ± 1.06	4.81 ± 1.10	4.69 ± 1.02	0.579
Triglyceride (mmol/L)	2.17 ± 1.50	2.38 ± 1.61	1.88 ± 1.30	0.095
LDL-c (mmol/L)	2.82 ± 0.92	2.88 ± 0.86	2.74 ± 1.00	0.425
HDL-c (mmol/L)	1.23 ± 0.29	1.22 ± 0.28	1.24 ± 0.31	0.644
UACR (mg/g)	18.0(7.8, 81.9)	13.4(6.7, 54.7)	44.5(10.4, 152.4)	0.006^∗∗^

Data are means ± SD, median (IQR), or *n* (%). CAN: cardiovascular autonomic neuropathy; BP: blood pressure; DSPN: distal symmetric polyneuropathy; HbA1c: glycated hemoglobin; LDL-c: low-density lipoprotein cholesterol; HDL-c: high-density lipoprotein cholesterol; UACR: urinary albumin-to-creatinine ratio. ^∗^*P* < 0.01 (non-CAN group vs. CAN group); ^∗∗^*P* < 0.01 (non-CAN group vs. CAN group); ^∗∗∗^*P* < 0.001 (non-CAN group vs. CAN group).

**Table 2:** The indicators of cardiovascular autonomic nervous function.

Variables	Non-CAN (*n* = 60)	CAN (*n* = 43)	*P* value
CARTs			
Deep breathing, *n* (% abnormal)	23(38.3)	42(97.7)	<0.001
30 : 15 ratio, *n* (% abnormal)	0(0)	14(32.6)	<0.001
Valsalva maneuver, *n* (% abnormal)	5(8.3)	32(74.4)	<0.001
Postural BP change, *n* (% abnormal)	0(0)	6(14.0)	<0.001
CARTs score	1.0(0.5, 1.5)	2.0(2.0, 3.0)	<0.001
COMPASS 31 score	12.9 ± 6.9	23.7 ± 9.8	<0.001
HRV indices			
SDNN (ms)	126.5 ± 21.6	82.8 ± 22.3	<0.001
PNN50 (%)	4.3(2.2, 9.0)	1.4(0.3, 3.7)	<0.001
RMSSD (ms)	25.5(20.0, 37.3)	19.0(13.0, 37.0)	0.010
LF (ms^2^)	386.1(227.7, 545.9)	108.7(37.0, 196.1)	<0.001
HF (ms^2^)	239.0(125.0, 378.5)	104.6(42.0, 170.0)	<0.001
LF/HF ratio	1.49(1.07, 2.68)	0.94(0.62, 1.63)	0.001

Data are means ± SD, median (interquartile range), or *n* (%). CAN: cardiovascular autonomic neuropathy; CARTs: cardiovascular autonomic reflex tests; 30 : 15 ratio: lying to standing; COMPASS 31: Composite Autonomic Symptom Score 31; HRV: heart rate variability; SDNN: the standard deviation of the NN intervals; PNN50: the percentage of adjacent NN intervals with a difference greater than 50 ms; RMSSD: the root mean square differences of successive NN intervals; LF: low-frequency power; HF: high-frequency power.

**Table 3:** Correlation between COMPASS 31 score, HRV indices, and CARTs.

Variables	CARTs score		COMPASS 31 score	
	*r*_s_	*P* value	*r*_s_	*P* value
COMPASS 31 score	0.547	<0.001	/	/
HRV indices				
SDNN (ms)	−0.722	<0.001	−0.397	<0.001
PNN50 (%)	−0.449	<0.001	−0.175	0.078
RMSSD (ms)	−0.284	0.004	−0.120	0.227
LF (ms^2^)	−0.637	<0.001	−0.306	0.002
HF (ms^2^)	−0.456	<0.001	−0.149	0.134
LF/HF ratio	−0.350	<0.001	−0.222	0.024

CARTs: cardiovascular autonomic reflex tests; COMPASS 31: Composite Autonomic Symptom Score 31; HRV: heart rate variability; SDNN: the standard deviation of the NN intervals; PNN50: the percentage of adjacent NN intervals with a difference greater than 50 ms; RMSSD: the root mean square differences of successive NN intervals; LF: low-frequency power; HF: high-frequency power.

**Table 4:** Comparison of diagnostic value among COMPASS 31 score and HRV indices.

Variables	Cutoff	Sensitivity (%)	Specificity (%)	PPV (%)	NPV (%)	AUC	Youden's index	Kappa value
COMPASS 31 score	>19.5	67.4	83.3	74.4	78.1	0.816	0.51	0.52
HRV indices								
SDNN (ms)	≤95	79.1	91.7	87.2	85.9	0.919	0.71	0.72
PNN50 (%)	≤1.4	58.1	86.7	75.8	74.3	0.746	0.45	0.46
RMSSD (ms)	≤16	44.2	88.3	73.1	68.8	0.648	0.33	0.34
LF (ms^2^)	≤131.4	65.1	96.7	93.3	79.5	0.865	0.62	0.65
HF (ms^2^)	≤183.5	83.7	61.7	61.0	84.1	0.747	0.45	0.43
LF/HF ratio	≤0.86	48.8	88.3	75.0	70.7	0.699	0.37	0.39
Combined model	/	90.7	86.7	83.0	92.9	0.958	0.77	0.75

Combined model: the combination of COMPASS 31 score, SDNN, and LF. COMPASS 31: Composite Autonomic Symptom Score 31; HRV: heart rate variability; SDNN: the standard deviation of the NN intervals; PNN50: the percentage of adjacent NN intervals with a difference greater than 50 ms; RMSSD: the root mean square differences of successive NN intervals; LF: low-frequency power; HF: high-frequency power.

## Data Availability

The raw data used to support the conclusions of this manuscript are available from the corresponding author, without undue reservation, to any qualified researcher.

## Supplementary Materials

Supplementary Materials Supplementary Table S1: comparison of the COMPASS 31 domain scores.
