# Supplementary material for: Combination of Composite Autonomic Symptom Score 31 and Heart Rate Variability for Diagnosis of Cardiovascular Autonomic Neuropathy in People with Type 2 Diabetes
Source: J Diabetes Res. 2020 Oct 30;2020:5316769. doi: 10.1155/2020/5316769 (PMC7648703; doi:10.1155/2020/5316769)
Supplement: Supplementary Materials — Supplementary Table S1: comparison of the COMPASS 31 domain scores. [file 5316769.f1.pdf]

Supplementary Information

Table S1 Comparison of the COMPASS 31 domain scores

COMPASS 31 domain scores	non-CAN （n=60）	CAN （n=43）	*P* value
orthostatic intolerance	0.0（0.0, 0.0）	8.0（0.0, 12.0）	<0.001
vasomotor	0.0（0.0, 0.0）	0.0（0.0, 1.7）	0.01
secretomotor	6.4（2.1, 8.6）	8.6（4.3, 8.6）	0.05
gastrointestinal	2.7（0.9, 4.5）	4.5（2.7, 7.1）	<0.001
bladder	1.1（0.0, 2.2）	2.2（1.1, 3.3）	0.001
pupillomotor	1.3（0.0, 1.7）	1.7（0.7, 2.3）	0.05

CAN, cardiovascular autonomic neuropathy; COMPASS 31, composite autonomic symptom score 31
